# Expert guidance for the rehabilitation of children with arthrogryposis: protocol using an integrated knowledge translation approach

**DOI:** 10.1186/s40900-022-00336-y

**Published:** 2022-02-18

**Authors:** Noémi Dahan-Oliel, Sarah Cachecho, Alicja Fąfara, Francis Lacombe, Ani Samargian, André Bussières

**Affiliations:** 1 Shriners Hospital for Children, Montreal, Canada; 2 School of Physical and Occupational Therapy, McGill University, Montreal, Canada; 3 Institute of Physiotherapy, Jagiellonian University, Kraków, Poland; 4 Centre de Réadaptation Lucie-Bruneau, Montreal, Canada; 5 AMCSupport Inc, Spartanburg, SC USA

**Keywords:** Arthrogryposis, Rehabilitation, Pediatrics, Clinical guidance, Consensus, Modified Delphi, Integrated knowledge translation

## Abstract

**Background:**

Arthrogryposis multiplex congenita (AMC) is a group of rare congenital disorders characterized by multiple joint contractures present at birth. Contractures can affect different body areas and impact activities of daily living, mobility and participation. Although early rehabilitation is crucial to promote autonomy and participation in children with AMC, empirical evidence to inform best practice is scarce and clinical expertise hard to develop due to the rarity of AMC. Preliminary research involving stakeholders in AMC (youth with AMC, parents, and clinicians) identified priorities in pediatric rehabilitation. Scoping reviews on these priorities showed a lack of high quality evidence related to rehabilitation in AMC. The objective of this project is to provide rehabilitation expert guidance on the assessment and treatment of children with AMC in the areas of muscle and joint function, pain, mobility and self-care, participation and psychosocial wellbeing.

**Methods:**

An integrated knowledge translation approach will be used throughout the project. Current rehabilitation practices in AMC will be identified using a clinician survey. Using the Grading of Recommendations, Assessment, Development and Evaluations framework (GRADE) approach, a panel of interdisciplinary expert clinicians, patient and family representatives, and researchers will develop expert guidance on the assessment and treatment for pediatric AMC rehabilitation based on findings from the scoping reviews and survey results. Consensus on the guidance statements will be sought using a modified Delphi process with a wider panel of international AMC experts, and statements appraised using the Appraisal of Guidelines for Research and Evaluation II (AGREE II) tool. Theoretical facilitators and barriers toward implementing clinical guidance into practice will be identified among rehabilitation clinicians and managers to inform the design of dissemination and implementation strategies.

**Discussion:**

This multi-phase project will provide healthcare users and providers with research-based, expert guidance for the rehabilitation of children with AMC and will contribute to family-centered practice.

## Plain English summary

Arthrogryposis multiplex congenita (AMC) is a group of rare disorders where a child is born with stiff joints. Joint stiffness can be present in different parts of the body, making it difficult for the child to move, to walk and to participate in activities. Rehabilitation interventions starting early in life are very important to maximize autonomy. AMC being rare, it is difficult for clinicians to develop experience with this population and there is little research specific to rehabilitation interventions in AMC. In a previous research project, a group of youth with AMC and their families identified five areas of priority for rehabilitation: muscle and joint function, pain, mobility and self-care, participation and psychosocial wellbeing. The purpose of this project is to provide guidance for the evaluation and treatment of children with AMC, in these priority areas. The project involves multiple steps. First, we will perform an online survey with international rehabilitation practitioners to learn about current evaluation and treatment approaches used with children with AMC. We will then bring together expert clinicians, patient and family representatives, and researchers to develop the guidance statements. Then, we will perform an online survey with a larger group of international experts to validate and finalize the guidance statements. Finally, we will interview rehabilitation clinicians and managers to find out about what could help them or limit them in integrating this guidance into their practice. This project will provide healthcare users and providers with research and expert-based guidance for the rehabilitation of children with AMC.

## Background

Rehabilitation in rare diseases is often under-represented in research due to lack of funding [1, 2], thereby limiting availability of evidence and resources for management. So is the case for arthrogryposis multiplex congenita (AMC) or arthrogryposis. AMC describes congenital joint contractures in two or more body areas [3], and affects between 1/3000 and 1/56,000 live births depending on the region surveyed, classification and coding used [4–6]. Individuals with AMC may have contractures in the upper limbs, lower limbs, the spine and jaw, with varying distribution and severity, causing limited joint movement and muscle weakness [7, 8]. Contractures do not progress to previously unaffected joints, but may change over time with growth and treatment [3]. Function in daily activities may be decreased in children with AMC, especially in the areas of self-care, transfers, mobility, and sports [9–12]. Children with AMC typically undergo several orthopedic surgeries to correct limb deformities, early and post-operative rehabilitation, splinting and bracing to improve range of motion [13, 14]. Early intensive rehabilitation is warranted [15], and advocated by many researchers [16]. However, rehabilitation providers reported a lack of knowledge and experience when treating individuals with AMC [17], given to the rarity and heterogeneity of AMC, and limited evidence as few studies have documented the rehabilitation treatment for this population.

**Fig. 1:**
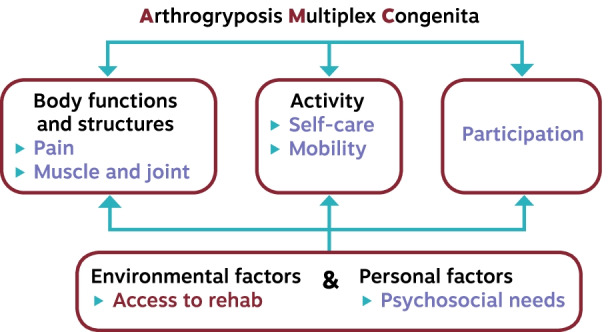
Mapping the rehabilitation priorities in AMC to the International Classification of Functioning, Disability and Health Framework

A recent qualitative study identified rehabilitation needs for youth with AMC, their parents, and clinicians through semi-structured interviews [17]. These needs included promoting independence in activities of daily life, access to rehabilitations services, addressing psychosocial needs, managing pain, and addressing physical needs. Further validation of these needs was conducted at the July 2017 annual AMC support group (AMCSI) meeting in Las Vegas. Twenty-four stakeholders (i.e., youth with AMC, parents, and clinicians) completed a questionnaire to rate the importance of the identified priorities and ranked their top five priorities. Using the World Health Organization’s International Classification of Health, Functioning and Disability (ICF) as a theoretical framework, the afore-mentioned rehabilitation needs were mapped to ICF domains (Fig. 1) [18]. This exercise led to following five priorities for the development of rehabilitation clinical guidance: muscle and joint function, pain, mobility and self-care, participation, and psychosocial wellbeing. Access to rehabilitation service including continuity of care from childhood to adulthood was considered as a transversal priority specific to each region and country’s policies.

Preliminary work consisted of a series of scoping reviews on the five priority areas as a lack of empirical studies in rehabilitation precluded a systematic review [8, 19–21]. These scoping reviews revealed a lack of high-quality studies (e.g., clinical trials) or consensus guidelines to guide clinicians toward the most clinically useful and suitable assessment tools and best evidence-based rehabilitation interventions for individuals with AMC. In light of the paucity of research to guide clinical decisions, the expertise, experience and knowledge of clinicians is important to inform best practice [22, 23]. Furthermore, the role of family representatives in rehabilitation research is essential to facilitate the research process and the application of the results, to create partnerships and ensure client- and family-centeredness [24, 25]. An integrated knowledge translation approach (iKT) will be used throughout this project. IKT is a collaborative model of research that includes knowledge users as research partners, such as healthcare professionals, patient and family representatives, and decision and policy makers [26]. Close collaboration between researchers and knowledge users provides a better understanding of the problem, the environment and context where the research will be used, as well as potential barriers to dissemination and implementation, and ensures the outcomes are in line with priorities of end users [26–28].

Developing rehabilitation expert guidance using the current literature and expert opinion addresses the important knowledge gap in this field. Expert guidance can inform key end-users, such as clinicians, youth, and family representatives, on the assessment tools and treatment indicated for this population. Thus, the overall aim of this project is to develop rehabilitation expert guidance, on the assessment and treatment of children with AMC to improve muscle and joint function, pain, mobility and self-care, participation, and psychosocial wellbeing.

## Methods

### Study design

The research team, composed of key stakeholders (i.e., youth with AMC and caregivers, expert clinicians, and researchers), is involved in all phases of this integrated knowledge translation (iKT) project. Members’ clinical expertise and lived experience ensures that the project is in line with the priorities and needs of end-users. The research team, including patient and clinician partners, has been meeting on a monthly basis to develop the research question and determine the methodology and targeted outcomes. The team will continue to meet monthly during the research process and members will be assigned some tasks to complete between meetings. Research partners will provide methodological and technical expertise and guidance in all steps of the study. Knowledge users will contribute in developing tools (i.e., questionnaires and surveys), identifying new partnerships and recruiting participants, interpreting findings, co-developing the expert guidance statements, and identifying strategies for knowledge dissemination [27]. This project is composed of the following five phases:

Phase 1: clinician survey to describe the current practice among rehabilitation clinicians working with children with AMC;

Phase 2: develop expert guidance statements for the rehabilitation management of children with AMC in the five priority areas listed previously;

Phase 3: validate and finalize the expert guidance statements using a modified Delphi-process with international clinical experts and family/patient representatives worldwide.

Phase 4: appraise the final expert guidance and evaluate the rigor and robustness of the methodology by external reviewers using the Appraisal of Guidelines for Research and Evaluation II (AGREE II) questionnaire [29];

Phase 5: identify the perceived facilitators and barriers to the uptake of the clinical guidance among rehabilitation professionals working with children with AMC, and their clinic managers to inform the development of knowledge translation strategies.

### Study procedures (Fig. 2)

**Fig. 2:**
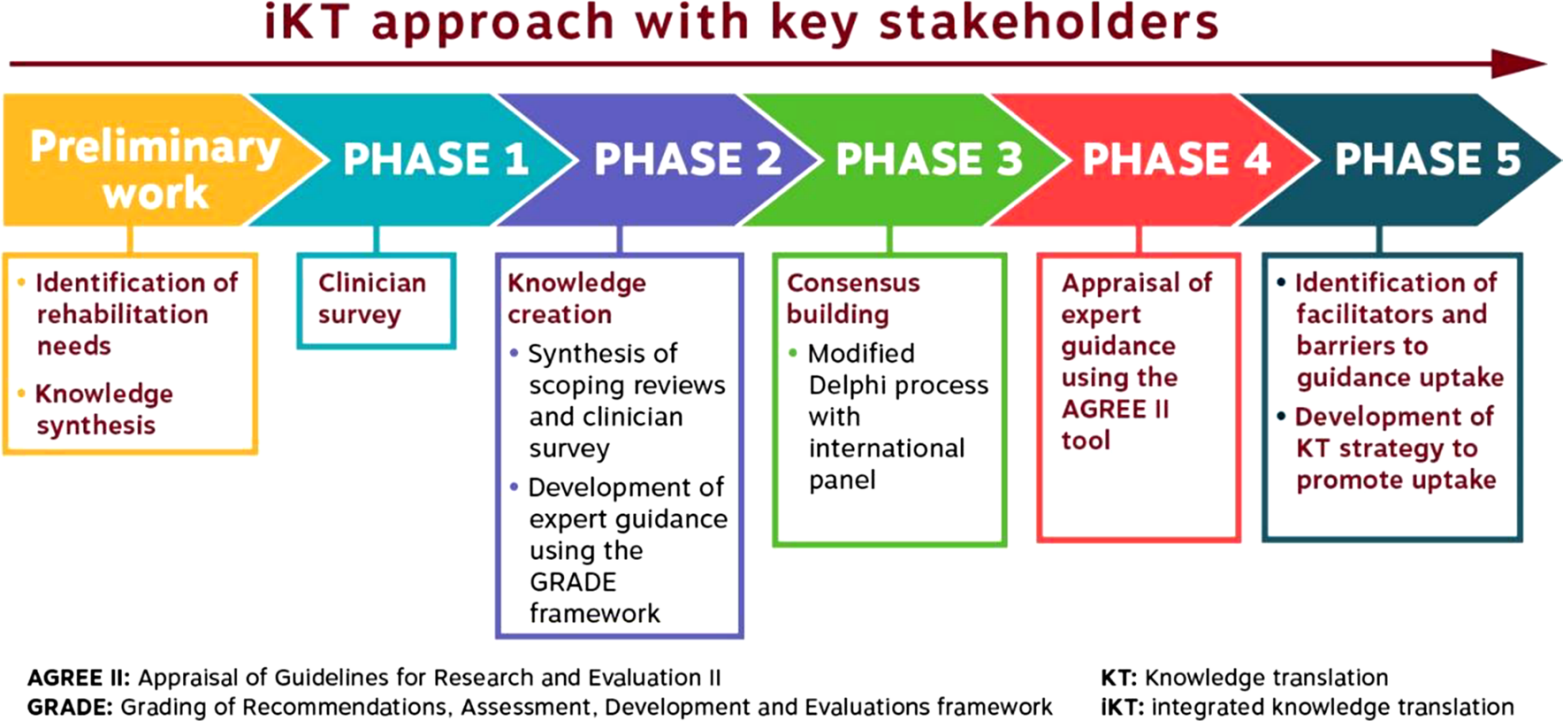
Integrated knowledge translation design and study flow

*Phase 1: Clinician survey* To inform current clinical practice in pediatric rehabilitation in AMC, the experience of rehabilitation practitioners (occupational therapists (OT, physical therapists (PT), social workers (SW), physiotherapy technologists) working with children with AMC worldwide will be sought through an electronic survey questionnaire. The survey will inquire about the different assessments and interventions that rehabilitation practitioners use with this population, in relation to the identified priority areas (i.e., muscle and joint function, pain, mobility and self-care, participation, and psychosocial wellbeing), for each age group (0–2 years, 3–6 years, 7–12 years, 13–18 years). The questionnaire will be based on the findings from the scoping reviews and from the experience of clinicians on the team, and include the assessment tools and treatment strategies reported in the literature [8, 19–21]. We will gather demographic information on the respondents (profession, country, years of experience working with children with AMC), and their e-mail address should they wish to be contacted for the subsequent phases of the project. The survey will be built on the Qualtrics platform, Shriners Hospital for children’s (SHC) clinical research survey platform, and will be piloted with one OT and one PT external to the research team, to identify any content and formatting issues. Invitations to participate will be sent electronically with a link to the survey. Potential participants will be recruited from hospitals and rehabilitation centers in North America. Participants around the world were identified through the literature review, from contacts established by the research team through previous research collaborations and from speakers at AMC annual conferences [30]. With this approach, the research team was able to identify over 300 potential participants (PTs, OTs, SWs and physiotherapy technologists) across all continents. A snowball recruitment technique will also be used to reach as many clinicians as possible worldwide. A recruitment flyer will be shared on social media channels of AMC Support Inc. In order to complete the survey, participants must have two years or more of experience working with children with AMC. Two reminders will be sent at 2 weeks interval, from the date the initial invitation was sent. Participants will be asked to provide electronic consent prior to completing the survey, with the consent discussion provided as an attachment in the invitation e-mail and as a PDF document at the beginning of the survey. Approval from the institutional review board of the Faculty of Medicine of McGill University was obtained (A03-E51-20B). Results from the survey will be analyzed quantitatively and qualitatively. The qualitative analysis will consist of summarizing participants’ comments for each section. Assessments and interventions used will be extracted and frequency counts for each will be conducted. Demographic information will be analysed using frequency counts.

*Phase 2: Developing expert guidance statements* The panel will be composed of three working groups including the research team (OT, PT, youth with AMC, parent of a child with AMC, researchers), clinician experts, and family representatives in AMC from various countries (e.g., Poland, United States, Australia, Norway, Spain). The clinician experts will be identified from previous contacts and through the clinician survey (Phase 1) and must have at least 5 years of experience working with children with AMC. One team will address muscle and joint function of the lower limbs and mobility, another team will address the upper limbs and self-care, and the third team will cover pain, participation and psychosocial wellbeing. The Grading of Recommendations, Assessment, Development and Evaluations framework (GRADE) will guide the process for the development of the guidance statements [31]. Preparatory documents including a summary of the literature and the clinician survey, as well as a summary of outcome measures to consider (description, population, scoring, psychometric properties, cost, training required) will be shared with the team member one week before the first meeting. Each group will meet remotely, on a weekly basis, for 5 weeks. Meetings will be led by an expert in the GRADE approach. The groups will consider findings from the scoping reviews [8, 19–21], and the results from the clinician survey (Phase 1) to guide the discussions regarding important outcomes pertaining to each priority area. Important questions and outcomes will be identified, and the balance of desirable (e.g., improved muscle and joint function, mobility, autonomy) and undesirable outcomes (e.g., fatigue, pain) will be considered. Aspects of suitability, feasibility, clinical relevance and costs of assessments and treatment interventions will be discussed when formulating the guidance statements. Each statement will be discussed extensively until the group reached consensus on content and wording. All discussion points will be documented in an Excel sheet and statements will then be transcribed on a word document along with a summary of the literature and group’s remarks. Both documents will be shared on screen during meetings and modified based on discussion. In order to achieve consensus, group members will vote verbally on each guidance statement (agree/disagree). Statements will be modified until consensus reaches 75% unanimity.

*Phase 3: Consensus building* In order to ensure the generalizability and acceptance of guidance statements, and to improve the likelihood of uptake in clinical practice, it is important to seek input from patient representatives and from a wide range of expert clinicians worldwide, across different health disciplines and working in different settings. Therefore, in order to reach consensus on the statements with a wider audience, a modified Delphi process will be used with a group of approximately 20 experts in the field of AMC who were not members of the panel in Phase 2. Invitations will be sent to larger number of potential participants in order to reach our goal of 20 participants. Those who completed the clinician survey and expressed an interest in participating in subsequent phases of the project will be invited. The modified Delphi method was selected as it overcomes geographical barriers and allows participants to remain anonymous and have equal opportunity to share without bias [22, 32, 33]. The modified Delphi process will consist of an online survey presenting the different expert guidance statements, with a summary of the literature and the expert opinion. For each statement, participants will have to rate their level of agreement on a 7-point Likert scale (from strongly disagree to strongly agree) and will be able to provide comments. Questions will include aspects of feasibility, clinical relevance, whether items should be included and the clarity of the wording used, and the format of how this information should be presented. After the first survey (i.e., round 1), results will be analyzed quantitatively and qualitatively. Statements that meet ≥ 80% agreement (i.e., 80% or more answered “agree” or higher) may be slightly modified based on comments from the participants. Statements that meet < 80% agreement will either be modified or eliminated, based on the comments from the participants. Items that meet ≥ 80% disagreement (i.e., 80% or more answered “disagree” or lower) will likely be eliminated unless comments from participants suggest otherwise. Results from the previous round will be summarized and sent with the modified guidance statements for a subsequent round. Only participants having completed the first round will be invited to participate in the following rounds. This is an iterative process and we expect three rounds of surveys until ≥ 80% agreement is met on all statements. Demographic data of participants (e.g., profession, country, years of experience) will be collected. The surveys will be e-mailed with a link to the Qualtrics platform. Participants will have three weeks to complete each survey with a reminder sent after two weeks. Should the participation rate in the second and subsequent rounds be less than 50% after three weeks from the time the survey is sent, up to two additional reminders, at two weeks intervals, will be sent. The deliverable of this phase will be a set of expert clinical guidance statements for rehabilitation of children with AMC.

*Phase 4: External review* Four to six independent reviewers, who did not participate in the previous phases, will be invited to complete the AGREE II questionnaire to appraise the final expert guidance and to ensure the rigor and robustness of the methodology [29]. Reviewers will consist of researchers or clinicians with either methodology expertise (i.e., in guideline development) or content expertise (i.e., rehabilitation or AMC). Feedback received will be collected and considered in a revised draft. The AGREE-II consists of 23 items divided into six domains (Score and purpose; Stakeholder involvement; Rigour of development; Clarity of presentation; Applicability; Editorial independence) and two global rating items. Items are rated on a 7-point scale (i.e., strongly disagree to strongly agree). Domain scores for the AGREE II will be calculated using the formula in the user manual, resulting in a percentage score [($\frac{obtained \, score-minimum \, possible \, score}{maximum \, possible \, score-minimum \, possible \, score}\times 100$)] [29]. Although there are no set minimum domain scores to differentiate between high- and poor-quality guidelines, a higher percentage indicates a better score for each domain. Items from the AGREE II that score below 3 will be reviewed and addressed by the research team.

*Phase 5: Identifying perceived facilitators and barriers to the uptake of clinical guidance & development of KT strategy* Theoretical factors (facilitators and barriers) regarding the uptake of pediatric rehabilitation guidance will be identified among approximately 13 rehabilitation practitioners working with the pediatric AMC clientele and about five pediatric musculoskeletal clinic managers identified through convenience sampling. The Theoretical Domains Framework (TDF), a framework used to identify influences on behaviour change, will be used for this phase to assess what factors likely influence healthcare professionals’ behaviour regarding the uptake of clinical practice guidelines [34–36]. The TDF consists of 84 theoretical constructs summarized into 14 domains, as depicted in Table 1*.* Data will be collected through individual interviews to cater to different schedules and time zones as we are reaching out to professionals worldwide. Demographic data will be gathered (profession, country, and years of experience working with children with AMC or working as a clinical manager with pediatric musculoskeletal clientele). The interview guide will be adapted from existing TDF interview topic guides [36–39]. On average, two to three questions per TDF domain with prompts are considered sufficient to cover all TDF domains [40]. The questions will be used to identify facilitators and barriers within each domain, exploring influences such as the participants’ local context of practice, patient and professional influences, and environmental constraints. Individual interviews will be conducted remotely using Microsoft Teams, recorded and then data will be transcribed verbatim and anonymized. Qualitative data analysis will be carried out by pairs using a coding guideline until consensus is achieved [40]. Data will be analyzed using a combination of deductive and inductive coding methods. Deductive coding will be performed first to generate the framework for a content analysis following a coding guideline based on the TDF domains. A coding guideline will be created by the research team to minimize subjective bias and ensure that the style of coding is consistent amongst all coders [40]. Participant citations will be attributed to the domain that best reflected its key theme. Inductive coding will be performed thereafter to further analyze the transcripts by attributing belief statements to each participant citation [40]. Once consensus has been reached by the pairs of independent coders, relevant TDF domains will be determined based on three criteria: (1) presence of beliefs that conflicted one another; (2) high frequency of utterances under specific belief(s) within a domain; (3) indication of strong beliefs that would impact the target behaviour.

**Table 1:** Domains of the Theoretical Domains Framework and definitions

Domain	Definition
Knowledge	An awareness of the existence of something (scientific, procedural, task environment)
Skills	An ability or proficiency acquired through practice
Social/Professional Role and Identity	A coherent set of behaviours and displayed personal qualities of an individual in a social or work setting
Beliefs About Capabilities	Acceptance of the truth, reality or validity about an ability, talent or facility that a person can put to constructive use
Optimism	The confidence that things will happen for the best or that desired goals will be attained
Beliefs About Consequences	Acceptance of the truth, reality, or validity about outcomes of a behaviour in a given situation
Reinforcement	Increasing the probability of a response by arranging a dependent relationship, or contingency, between the response and a given stimulus
Intentions	A conscious decision to perform a behaviour or a resolve to act in a certain way
Goals	Mental representations of outcomes or end states that an individual wants to achieve
Memory, Attention and Decision Processes	The ability to retain information, focus selectively on aspects of the environment and choose between two or more alternatives
Environmental Context and Resources	Any circumstance of a person’s situation or environment that discourages or encourages the development of skills and abilities, independence, social competence and adaptive behaviour
Social Influences	Those interpersonal processes that can cause individuals to change their thoughts, feelings, or behaviours
Emotion	A complex reaction pattern, involving experiential, behavioural, and physiological elements, by which the individual attempts to deal with a personally significant matter or event
Behavioural Regulation	Anything aimed at managing or changing objectively observed or measured actions

The identified relevant TDF domains and the corresponding identified facilitators and barriers will be matched with behaviour change techniques using a matrix by Michie and colleagues [41]. This matrix provides 35 behavior changing techniques and indicates their effectiveness in changing each construct domain of the TDF. These identified behaviour change techniques will help to inform knowledge translation strategies to promote behaviour change regarding the uptake of the clinical practice guidelines. A knowledge translation panel comprised of 10–12 members including rehabilitation professionals and managers, patients and families, experts in knowledge translation and health psychology and implementation science will meet over 2 separate 60–90 min panel meetings in order to take the behavior change technique findings and findings from the literature to brainstorm potential KT interventions to overcome the modifiable barriers and enhance the facilitators promoting behavior change. The final knowledge translation strategies will be selected based on their effectiveness and feasibility to implement.

## Discussion

This multi-phase project uses an iKT approach to guide the development of rehabilitation expert guidance for children with AMC, based on the needs and expertise of key stakeholders, including clinicians, youth and families with AMC, as well as decision makers, and contributes to family-centered practice [26]. An iKT approach is beneficial to optimize the uptake of the new knowledge into practice and in decision making [26–28, 42]. Despite limited evidence to support specific iKT strategies, a scoping review on iKT use in healthcare identified facilitators, barriers and outcomes to consider in the research process [42]. Ensuring a clear understanding of goals, roles and expectations, fostering open communication and using a common language, and having regular opportunities to exchange throughout a project, are a few enablers, among many others, that will promote the continuity and sustainability of knowledge users’ engagement in the research process [24, 42].

This project will help synthesize the existing knowledge and current practice in pediatric rehabilitation in AMC, identify gaps, create consensus-based expert guidance needed to inform clinical decisions for AMC, and identify perceived facilitators and barriers to the uptake of clinical guidance in practice to guide KT strategies. The results will provide healthcare users and providers with research-based, expert guidance to address physical, activity, participation and psychosocial needs of children with AMC. The project addresses one of the research and clinical priorities articulated during the 2^nd^ and 3^rd^ International Symposia on arthrogryposis [43, 44], specifically the development of guidelines for clinical care of AMC. The symposia brought together an international group of clinical experts, patients and families.

Although input from international stakeholders will be sought for this project, the applicability of the clinical guidance may be limited by regional and cultural factors. Since AMC is an umbrella term including many different diagnoses with a variety of clinical presentations and severities [7, 8], certain guidance statements may not be applicable across all diagnoses. In order to assist knowledge users to understand possible nuances and adapt the guidance to their and their client’s reality, clarifications and remarks will accompany the guidance statements. In addition, guidance statements will have to be revised and updated in the future to reflect advances in research and practice.

## Data Availability

Not applicable.

## Abbreviations

AGREE II: Appraisal of Guidelines for Research and Evaluation II
AMC: Arthrogryposis multiplex congenital
AMCSI: AMC support group
GRADE: Grading of Recommendations, Assessment, Development and Evaluations framework
ICF: International Classification of Health, Functioning and Disability
iKT: This integrated knowledge translation
OT: Occupational therapist
PT: Physical therapists
SW: Social workers
SHC: Shriners Hospital for children
TDF: Theoretical Domains Framework
